# Breed‐specific vertebral heart score, vertebral left atrial size, and radiographic left atrial dimension in Cavalier King Charles Spaniels: Reference interval study

**DOI:** 10.1111/vru.13036

**Published:** 2021-11-18

**Authors:** Mara Bagardi, Chiara Locatelli, Martina Manfredi, Jessica Bassi, Carlotta Spediacci, Sara Ghilardi, Davide D. Zani, Paola G. Brambilla

**Affiliations:** ^1^ Dipartimento di Medicina Veterinaria Università degli Studi di Milano Lodi Italy

**Keywords:** breed‐specific heart scale, Cavalier King Charles Spaniel, radiographic atrial measurements, vertebral heart scale

## Abstract

Cavalier King Charles Spaniels (CKCS) are predisposed to developing myxomatous mitral valve disease (MMVD), with radiographs frequently used to screen for evidence of left‐sided cardiomegaly secondary to MMVD. Vertebral heart size (VHS), vertebral left atrial size (VLAS), modified VLAS (M‐VLAS), and radiographic left atrial dimension (RLAD) are reported as objective measurements of global heart size and left atrial size. Normal VHS in CKCS (10.6 ± 0.5) is reportedly higher than the non‐breed‐specific value (9.7±0.5). Breed‐specific VLAS, M‐VLAS, and RLAD cut‐offs have not been reported in CKCS. The aim of this prospective reference interval study was to describe the VHS, VLAS, M‐VLAS, and RLAD values for 30 clinically healthy adult CKCS. Inclusion criteria were unremarkable physical examination, normal echocardiography, and thoracic radiographs without malposition/abnormalities. There were 22 female and eight male dogs. Ages ranged from 1 to 6 years. The VHS mean value in our sample was 10.08 ± 0.56 (95% range, 9.87‐10.29). This was significantly greater than a previously published general canine reference value of 9.7 ± 0.5 and significantly less than a previously published CKCS breed‐specific value of 10.6 ± 0.5 (*P* < 0.01). Mean VLAS, M‐VLAS, and the RLAD values in our study were 1.79 ± 0.3 (95% range, 1.68‐1.9), 2.23 ± 0.44 (95% range, 2.06‐2.39), and 1.2 ± 0.34 (95% range, 1.07‐1.33), respectively. These were significantly less than previously published reference interval values (*P* < 0.001). The VHS, M‐VLAS, and the RLAD were not affected by sex, body weight, or BCS; whereas the VLAS was moderately affected by body weight. Findings from this study can be used as background for future thoracic radiographic assessments in CKCS.

**Abbreviations**
ACVIM: American college of veterinary internal medicine
BCS: body condition score
CKCS: Cavalier King Charles Spaniels
DV: dorso‐ventral view
E/A: E and A waves ratio
LA: left atrium
LA/Ao: left atrium to aorta ratio
LA/Ao_Sx: short‐axis left atrium indexed to the short‐axis aortic root
LAD/AoD_Lx: long‐axis left atrial dimension indexed to the long‐axis aortic valve annulus diameter
LL: left lateral view
LV: left ventricle
LVIDD: left ventricular internal diameter at end‐diastole
LVIDDN: normalized left ventricular internal diameter in diastole
LVIDS: left ventricular internal diameter at end‐systole
MMVD: myxomatous mitral valve disease
M‐VLAS: modified vertebral left atrial size
MVP: mitral valve prolapse
RL: right lateral view
RLAD: radiographic left atrial dimension
T4: fourth thoracic vertebra
TD/TW: thoracic depth – thoracic width ratio
v: vertebral unit
VHS: vertebral heart score
VLAS: vertebral left atrial size.

## 1 INTRODUCTION

Myxomatous mitral valve disease (MMVD) is a common cardiovascular disease affecting dogs, progressing to mitral regurgitation, and eventually heart failure.^1^ The incidence is age‐related and is particularly high in breeds such as the Cavalier King Charles Spaniels (CKCS).^1^, ^2^ Fifty percent of CKCS are affected by the age of 6‐7 years, and almost 100% are affected by the age of 11.^1^, ^2^ In this breed, thoracic radiographs are frequently used to screen for evidence of left‐sided cardiomegaly secondary to MMVD.^3^, ^4^ Published radiographic measures of heart size include vertebral heart size (VHS), vertebral left atrial size (VLAS), its modified version (M‐VLAS), and radiographic left atrial dimension (RLAD).^3^, ^5^, ^6^, ^7^, ^8^, ^9^ The VHS is influenced by different morphotypes and several studies have described breed‐specific reference range.^10^, ^11^, ^12^, ^13^, ^14^, ^15^, ^16^, ^17^, ^18^, ^19^, ^20^ Only normal values of VHS in CKCS have been investigated and are reported to be higher than not‐breed‐specific reference ranges (respectively 10.6 ± 0.5 vs 9.7 ± 0.5).^3^, ^10^

The VLAS, M‐VLAS, and RLAD have recently been proposed as new radiographic methods for quantifying left atrial size in dogs^7^, ^8^, ^9^; however, no published studies have evaluated VLAS, M‐VLAS, and RLAD in different breeds. A recent study reported values for VLAS in Chihuahuas.^21^ Breed‐specific VLAS, M‐VLAS, and RLAD reference values have not been reported for CKCS. The 2019 American College of Veterinary Internal Medicine (ACVIM) MMVD consensus statement recommends, if available, the use of breed‐specific radiographic normal values.^22^

The first aim of this study was to describe breed‐specific reference interval values for VHS, VLAS, M‐VLAS, and RLAD in a sample population of healthy adult CKCS. The second aim was to test the hypothesis that these radiographic measures of cardiac size in CKCS would differ from previously published reference values. The third aim was to test the hypotheses that radiographic measures of cardiac size in CKCS would not be significantly affected by sex, body condition score (BCS), or chest conformation; but would be significantly affected by recumbency.

## 2 MATERIALS AND METHODS

This study was a prospective, single‐center, reference interval study design. Thirty, healthy CKCS referred for preoperative evaluation or cardiologic screening were recruited from June 2019 to March 2021 at the Veterinary Medicine Department of the University of Milan. The sample size was based on convenience sampling.

### 2.1 Ethical approval

This study being a part of a larger project, informed consent was obtained from all the clients before starting any examination procedures for included dogs. The radiographic examinations were carried out as per the hospital's standard procedures.

### 2.2 Case selection

All included CKCS underwent a complete physical examination, thoracic radiography, and echocardiography. Dogs were considered healthy based on the absence of prior clinical conditions/abnormalities documented by the owners and unremarkable physical examination, cardiovascular assessment, and transthoracic echocardiogram.^21^ Dogs with cardio‐structural heart diseases and cardiac chamber enlargement identified on echocardiogram were excluded from the study, as well as dogs aged <12 months because of the possible influence of young age and skeletal immaturity on the radiographic vertebral‐based measurements. Thoracic radiographs that revealed an overt malposition of the patient (as the considerable rotation, the movement due to excessive breathing for tachypnea or the brachial muscles superimposition on the cranial aspect of the thorax) or the presence of thoracic vertebral abnormalities (eg, hemivertebrae) were not included in the study.^23^, ^24^ All decisions for dog inclusion or exclusion were made by two veterinarians with more than fifteen years of clinical experience in veterinary radiology (D.Z.) and veterinary cardiology (C.L.), respectively, based on a consensus opinion.

### 2.3 Echocardiographic examination

All echocardiographic examinations were performed in unsedated dogs by a third‐year PhD veterinary cardiology student (M.B.) under the supervision of a veterinarian with more than 15 years of clinical experience in veterinary cardiology (C.L.), using the same ultrasonographic unit [MyLab50 Gold cardiovascular ultrasound machine (Esaote, Genova, Italy)] equipped with multi‐frequency phased array probes (3.5–5 and 7.5–10 MHz), chosen according to the weight of the subject. Each dog underwent a complete echocardiographic examination, which included transthoracic two‐dimensional, M‐mode, and Doppler imaging.^25^ An average of three cardiac cycles was used for each measurement. Left ventricular internal diameter at end‐diastole (LVIDD) and left ventricular internal diameter at end‐systole (LVIDS) were measured on two‐dimensional‐guided M‐mode right parasternal short axis images. End‐diastole of the left ventricle (LV) chamber was defined as the internal dimension at the onset of the QRS complex on the echo‐timing ECG. The end‐systolic LV chamber internal dimension was defined as the minimum chamber dimension. The measurements were made from inner edge (blood‐tissue interface) to inner edge. LVIDD was normalized to body size (LVIDDN) as previously described, and left ventricular diastolic dimension was considered normal with a LVIDDN <1.7.^22^, ^26^ Echocardiographic left atrial (LA) size was determined using two different body size‐indexed linear measurements: (a) short‐axis LA indexed to the short‐axis aortic root (LA/Ao_Sx) and (b) long‐axis left atrial dimension indexed to the long‐axis aortic valve annulus diameter (LAD/AoD_Lx). Short‐axis LA indexed to the short‐axis aortic root was measured by the two‐dimensional right parasternal short axis view as previously described.^27^ Short‐axis LA indexed to the short‐axis aortic root was calculated from left atrial and aortic root diameters. Left atrial and aortic root dimensions were measured from inner edge to inner edge, timed after the end of the T wave, in the earliest frame in which the aortic valve cusps were closed. For the evaluation of LAD/AoD_Lx, maximum long‐axis left atrial dimension was determined from a right parasternal long‐axis four chamber view where a line is drawn from the mid atrial septum, that is, region of the fossa ovale, to the internal reflection of the bright pericardium in the far field. This bisected the long axis LA area and is approximately parallel to the mitral annulus.^28^, ^29^ Left atrial size was considered normal when the LA/Ao_Sx was < 1.6 and the LAD/AoD_Lx was < 2.4.^22^, ^29^ The operator performing the echocardiographic measurements was blinded to the VHS, VLAS, M‐VLAS, and RLAD measurement. Only CKCS belonging to ACVIM class A were included in the study.^30^, ^31^

### 2.4 Radiographic examination

Radiographic examinations and measures were performed by a third‐year veterinary radiology PhD student (M.M.) supervised by a veterinarian with more than fifteen years of experience in veterinary radiology and clinical practice (D.Z.). They were blinded to echocardiographic findings. The assessment of the interobserver variability of VHS, VLAS, M‐VLAS, and RLAD by multiple observers was not the intent of the study and was previously evaluated.^23^ The radiographic examination included right lateral (RL), left lateral (LL), and dorso‐ventral (DV) views. The dogs were conscious and carefully contained to prevent an abnormally positioned thoracic vertebral column and trachea. All radiographs were acquired at the time of full inspiration. The thoracic radiographic studies were obtained with a digital system (RX D‐VET G35i, FUJIFILM Italia S.P.A., Milano, Italia) and radiographic exposure factors for each dog were based on patient body size.

A dedicated image analysis workstation (iMac Retina 5K, 27‐inch, 2014 with OsiriX© MD v. 8.0.2, Pixmeo SARL, Swizerland) was used for all radiographic measurements. The radiographic evaluations of VHS, VLAS, M‐VLAS, and RLAD were performed using digital calipers for both RL and LL radiographic views.^6^, ^7^, ^8^, ^9^, ^32^ Each length was expressed in vertebral body units (v) to the nearest 0.1 vertebra for all the described radiographic measurements.

**FIGURE 1:**
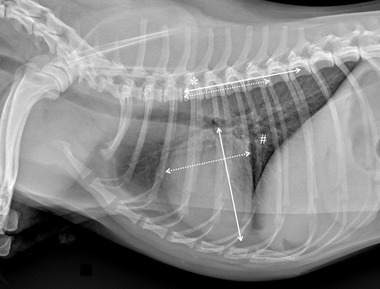
Right lateral thoracic radiographic image of a CKCS demonstrating the radiographic measurements of vertebral heart size (VHS) performed in this study (kVp 75, mAs 2.5). A line was drawn from the central and ventral border of the carina to the most distant point of the cardiac apex (solid line). The short‐axis (dotted line) line was measured at the widest part of the cardiac silhouette within the central one‐third region, typically near the ventral border of the caudal vena cava (#), and perpendicular to the long‐axis. The measurements of the two axes were then indexed to the thoracic vertebral bodies starting at the cranial edge of T4 (*) and summed (9.6 vertebrae in this example)

**FIGURE 2:**
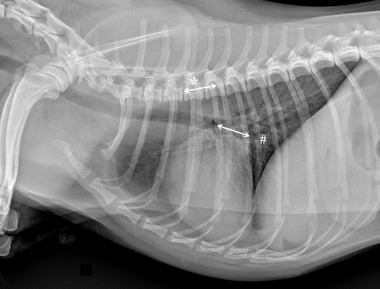
Right lateral thoracic radiographic image of a CKCS demonstrating the radiographic measurements of vertebral left atrial size (VLAS) perfomed in this study (kVp 75, mAs 2.5). A line was drawn from the central and ventral border of the carina to the caudal most border of the left atrium, where it intersected with the dorsal border of the caudal vena cava (#). This line was indexed to the thoracic vertebral bodies starting at the cranial edge of T4 (*) and summed (1.7 vertebrae in this example)

**FIGURE 3:**
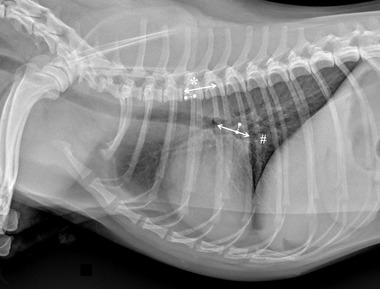
Right lateral thoracic radiographic image of a CKCS demonstrating the radiographic measurements of modified version of vertebral left atrial size (M‐VLAS) performed in this study (kVp 75, mAs 2.5). An initial line (solid line) was drawn from the center of the most ventral aspect of the carina to the intersection between the most caudal aspect of the left atrium and the dorsal border of the caudal vena cava (#). A second additional line (dotted line) was then drawn from the most distal border of the left atrium towards the first line, intersecting it perpendicularly. Two separate straight lines corresponding to the lengths of the first 2 lines were then drawn from the cranial edge of the T4 (*) and summed (2 vertebrae in this example)

**FIGURE 4:**
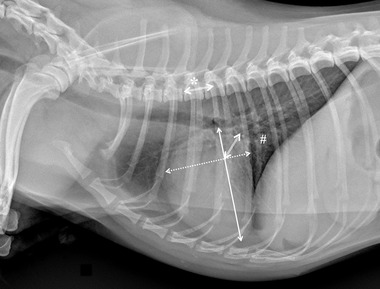
Right lateral thoracic radiographic image of a CKCS demonstrating the radiographic measurements of radiographic left atrial dimension (RLAD) performed in this study (kVp 75, mAs 2.5). The computer software was used to ensure a line (double line) bisecting the 90° angle formed by the intersection of the VHS long (solid line) and short (dotted line) axes connecting this point with the radiographic projection of the dorsal edge of the LA, both for RL and LL projection. These length was then drawn starting from the cranial edge of T4 (*), summed, and used as RLAD (1.4 vertebrae in this example)

**FIGURE 5:**
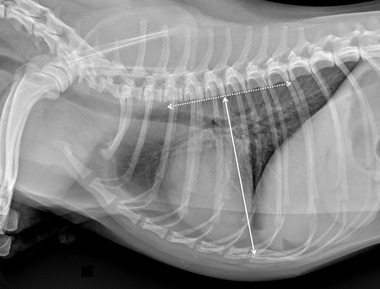
Right lateral thoracic radiographic image of a CKCS demonstrating the radiographic measurements of thoracic depth (solid line) measured from xiphoid process to the perpendicular of vertebral column in right lateral recumbency (kVp 75, mAs 2.5)

**FIGURE 6:**
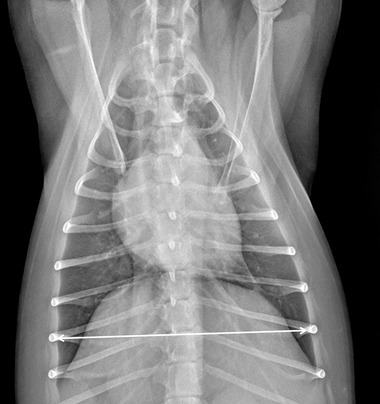
Dorso‐ventral thoracic radiographic image of a CKCS demonstrating the radiographic measurements of thoracic width measured as the distance between medial borders of eighth rib at their most lateral curvatures in dorso‐ventral recumbency (kVp 75, mAs 2.5)

Specifically for VHS, the long‐axis dimension (L) was measured from the ventral border of the largest of the main stem bronchi seen in cross section to the most ventral point of the cardiac apex. The short‐axis dimension (S) was drawn perpendicular to the long‐axis dimension from the caudal border of the cardiac silhouette at the dorsal aspect of the caudal vena cava to the cranial border of the cardiac silhouette. The two lengths (L and S) were then repositioned over the thoracic vertebrae, parallel to the vertebral canal, beginning to the fourth thoracic vertebrae (T4). The VHS was the sum of the two lines in vertebral body units (Figure 1). For VLAS, the length between the center of the most ventral aspect of the carina to the caudal aspect of the left atrium at point of intersection with the dorsal border of the caudal vena cava was measured. A line equal in length to this measurement was drawn from the cranial border of the T4 and extended caudally parallel to the vertebral canal. The reported VLAS was the length of this line in vertebral body units (Figure 2). Modified VLAS (M‐VLAS) was calculated starting from VLAS, as originally described,^7^ and a second dimensional measurement was made by placement of the digital caliper at the most distal LA border excluding the pulmonary vein orifice and extended to perpendicularly intersect with the first line. As before, the line was transposed on the vertebral column from the cranial edge of the T4 body and the M‐VLAS was defined as length of this measurement in vertebral body units (Figure 3).^8^ The computer software was used to ensure a line bisecting the 90° angle formed by the intersection of the VHS L and S axes connecting this point with the radiographic projection of the dorsal edge of the LA, both for RL and LL projection. This length was then drawn starting from the cranial edge of T4 and used as RLAD (Figure 4). In cases where it was difficult to differentiate the dorsal anatomical boundaries of the LA and the neighboring pulmonary veins, the most dorsal aspect of the soft tissue opacity seen at this level was routinely used for all measurements.^9^ Thoracic conformation was determined from the TD/TW ratio, as described by Buchanan and Bücheler.^6^ The depth of thorax was measured in the RL radiographic view from the cranial edge of xiphoid process to the ventral border of vertebral column along a line perpendicular to vertebral column (Figure 5). The width of the thorax was measured on a DV radiograph as the distance between medial borders of eighth ribs at their most lateral curvatures (Figure 6).^17^

[Correction added on 29 March 2022, after first online publication: The citations for Figure 1–6 have been corrected in this version.]

### 2.5 Statistical analysis

Statistical analyses were performed by a veterinarian with coursework training in statistics (C.L.) using commercially available statistics software (SPSS^TM^ 27.0, IBM, SPSS, USA). Descriptive statistics were generated. The distribution of data for continuous variables was assessed for normality by means of the Kolmogorov‐Smirnov test. Variables were normally distributed, and results were reported as mean ± standard deviation (SD) unless otherwise specified. A paired Student's *t*‐test was used to compare mean VHS, VLAS, M‐VLAS, and RLAD values between right and left lateral recumbency. An unpaired Student's *t*‐test was used to compare mean VHS, VLAS, M‐VLAS, and RLAD values between males and females, between dogs with different TD/TW ratios, particularly TD/TW higher or lower than 0.9, and between dogs with different BCS and body weights. The TD/TW 0.9 cut off was arbitrarily chosen due to the CKCS’ intermediate thoracic conformation. A Pearson correlation coefficient (*r*) was applied to study the correlation between all radiographic measurements, clinical (age, weight, BCS) and echocardiographic data (LVIDDN, LA/Ao_Sx, LAD/AoD_Lx, sphericity index). The correlation was considered weak, moderate, strong, or perfect respectively when the value of the correlation coefficient was 0.1‐0.3, 0.4‐0.6, 0.7‐0.9, or 1.^33^ A correlation analysis between VHS, VLAS, M‐VLAS, RLAD, and TD to TW ratio was performed to determine whether chest conformation was responsible for variation in these radiographic parameters. To compare obtained VHS, VLAS, M‐VLAS, and RLAD measurements, the values from Malcolm's study expressed as median and interquartile range were converted to mean and standard deviation (2.07 ± 0.25 vertebrae);^7^ the same was for M‐VLAS (2.6 ± 0.3 vertebrae) and RLAD (1.97 ± 0.57 vertebrae).^8^, ^9^, ^34^ A one sample *t*‐test was then used to test whether the VHS, VLAS, M‐VLAS, and RLAD in our population of CKCS differed from the mean reference values proposed by literature.^6^, ^7^, ^8^, ^9^, ^32^ A *P*‐value < 0.05 was considered significant for all analyses.

## 3 RESULTS

There were 22 of 30 (73.3%) females (n. 3 neutered) and eight of 30 (26.7%) males (n. 1 neutered); with a mean age of 2.66 ± 1.42 years (range: 1‐6 years) and a mean body weight of 7.92 ± 1.68 kg (range: 5.1‐12 kg). Twelve subjects (40%) weighed more than the proposed breed standard (5‐8 Kg). The mean BCS was 5/9 ± 1/9 (range: 4/9‐7/9). Nineteen dogs (63.33%) had Blenheim coat color type, n.1 (3.33%) ruby, n. 3 (10%) black and tan, and n. 7 (23.34%) tricolor. The small number of subjects with coats other than Blenheim did not permit statistical analysis against this physical parameter. All the clinical and the echocardiographic data are reported in Table 1.

**TABLE 1:** Clinical and echocardiographic data of all included healthy CKCS

	Overall population	Females	Males
N. of dogs	30	22	8
Sex	22F (3NF) 8 M	22 F 3NF	8 M
Age (years)	2.66 ± 1.42	2.79 ± 1.42	2.32 ± 1.47
Weight (kg)	7.92 ± 1.68	7.74 ± 1.67	8.42 ± 1.72
BCS	5/9 ± 1/9	5/9 ± 1/9	5/9 ± 1/9
Coat color type	19 Blenheim 1 Ruby 3 Black and tan 7 Tricolor	14 Blenheim 1 Ruby 2 Black and tan 4 Tricolor	5 Blenheim 1 Black and tan 3 Tricolor
LA/Ao_Sx	1.20 ± 0.17 1.22	1.22 ± 0.16^*^	1.07 ± 0.11
LAD/AoD_Lx	2.01 ± 0.19	2.08 ± 0.13^*^	1.81 ± 0.19
LVIDDN	1.30 ± 0.16	1.28 ± 0.16	1.33 ± 0.18
SI	1.57 ± 0.2	1.55 ± 0.21	1.65 ± 0.17

Abbreviations: F, females; NF, neutered females; M, males; BCS, body condition score; LA/Ao_Sx, short‐axis left atrium indexed to the short‐axis aortic root; LAD/AoD_Lx, long‐axis left atrial dimension indexed to the long‐axis aortic valve annulus diameter; LVIDDN, normalized left ventricular internal diameter in diastole; SI, sphericity index.

^*^Parameters significantly higher in females compared to males (*P* < 0.05).

In our study, the CKCS had a significantly lower VHS (10.08 ± 0.56; 95% range 9.87‐10.29) than the reference value of 10.6 ± 0.5 established by Lamb et al in 2001 for this breed (*P* = 0.002) and higher than the reference value of 9.7 ± 0.5 proposed by Buchanan and Bücheler in 1995 (*P* < 0.001).^6^, ^10^ The VLAS, M‐VLAS, and the RLAD of CKCS in our study were respectively 1.79 ± 0.3 (95% range, 1.68‐1.9), 2.23 ± 0.44 (95% range, 2.06‐2.39), and 1.2 ± 0.34 (95% range, 1.07‐1.33). These were less than the values previously reported by Malcolm et al (2.07± 0.25; *P* = 0.000), Lam et al (2.6 ± 0.3; *P* = 0.000), and Salguero et al (1.97 ± 0.57; *P* = 0.000).

Table 2 reports the radiographic values of the VHS, VLAS, M‐VLAS, and RLAD in RL and LL view from our study. No significant differences in VHS were found between LL and RL recumbencies (P = 0.25), whereas VLAS, M‐VLAS, and RLAD were significantly higher in LL than RL view (*P* < 0.001, *P* = 0.001, and *P* = 0.02, respectively). Both RL and LL VHS, VLAS, M‐VLAS, and RLAD did not significantly differ between males and females (*P* > 0.05). All radiographic measurements did not significantly differ between different BCS groups (*P* > 0.05). Mean TD to TW ratio was 0.91 ± 0.08 (95% range 0.88‐0.94). Fifteen dogs (50%) had a TD/TW ratio < 0.9 and n. 15 (50%) > 0.9. All CKCS of this study had an intermediate chest conformation (0.75 < TD/TW < 1.25).^6^ Thoracic depth to thoracic width ratio did not differ significantly between the sexes (*P* = 0.26).

**TABLE 2:** Vertebral heart score, vertebral left atrial size, modified vertebral left atrial size and radiographic left atrial dimension in 30 healthy Cavalier King Charles Spaniels

	Recumbency	N	Mean ± SD	95% Range	*P*
VHS	RL	30	10.08 ± 0.56	9.87‐10.29	0.25
	LL	30	10.00 ± 0.41	9.85‐10.17	
VLAS	RL	30	1.79 ± 0.3	1.68‐1.90	0.000
	LL	30	1.99 ± 0.25	1.90‐2.09	
M‐VLAS	RL	30	2.23 ± 0.44	2.06‐2.39	0.001
	LL	30	2.48 ± 0.28	2.38‐2.59	
RLAD	RL	30	1.20 ± 0.34	1.07‐1.33	0.02
	LL	30	1.37 ± 0.20	1.29‐1.44	

Abbreviations: VHS, vertebral heart score; VLAS, vertebral left atrial size; M‐VLAS, modified vertebral left atrial size; RALD, radiographic left atrial dimension; RL, right lateral view; LL, left lateral view.

The VHS, VLAS, M‐VLAS, and RLAD showed no correlation with BCS (*P* > 0.05), and body weight (*P* > 0.05). Only LL VLAS showed a moderate positive correlation with BCS (*r* = 0.38, *P* = 0.037). There was no significant correlation between the type of chest and VHS, VLAS, M‐VLAS, and RLAD in all included dogs and no significant differences were observed for dogs with different TD/TW ratio (higher and lower than 0.9). Conversely, CKCS with TD/TW ratio lower than 0.9 had greater LA/Ao_Sx ratio and lower sphericity index (both *P* = 0.001).

## 4 DISCUSSION

The purpose of this study was to describe breed specific reference values for VHS, VLAS, M‐VLAS, and RLAD in healthy adult CKCS.^6^, ^7^, ^8^, ^9^ Based on the authors’ review of the literature, this is the first published study proposing the reference intervals in this breed for VLAS, M‐VLAS, and RLAD. In this CKCS sample, the VHS was significantly higher than the not‐breed specific reference values initially established by Buchanan and Bücheler in 1995,^6^ but significantly less than the breed standard proposed by Lamb et al in 2001.^10^ In the study by Buchanan and Bücheler,^6^ there were no significant differences between RL and LL recumbencies for VHS. This is in accordance with our results. Lamb et al^10^ in 2001 evaluated only RL view in their study with multiple breeds, but Greco et al^35^ in 2008 reported a higher VHS value by 0.3 vertebra in RL recumbency compared to LL. Similarly, other studies found a higher VHS in RL recumbency than in LL recumbency.^12^, ^15^, ^17^, ^18^ Disagreement between studies may be explained first by differences in thoracic morphotypes among breeds. It was also hypothesized that the larger VHS in RL recumbency may be due to the divergent X‐ray beam and the larger distance of the heart from the cassette in RL recumbency.^35^ In addition, possible variations in radiographic cardiac size during the cardiac cycle (diastolic vs systolic dimensions) need to be considered. In fact, while respiratory cycle can be controlled when radiographs are taken, cardiac cycle cannot.^36^, ^37^ The previously reported mean VHS ± SD ranges from 9.9 ± 0.8 to 10.4 ± 0.8 vertebrae between end‐diastolic and end‐systolic measurements with fluoroscopy at peak inspiration for dogs positioned in right lateral recumbency.^37^ On average, mean VHS ± SD is 0.3 ± 0.3 vertebrae greater in diastole than in systole at peak inspiration, with VHS varying up to 0.97 vertebral units over the cardiac cycle in some individuals.^36^ Similar influence of cardiac cycle is observed on VLAS but however the same has never been described for RLAD.^37^ Furthermore, without further studies, we can only deduce that M‐VLAS, being a derivative of VLAS, might also show similar influence by cardiac cycle. Vertebral left atrial size, M‐VLAS, and RLAD in our study were significantly higher in LL than RL view. This can be anatomically justified by a possible overlapping of the venous sinus of the cava veins, of the coronary venous sinus and of the caudal vena cava outflows.

Buchanan and Bücheler did not detect any differences in the VHS between males and females.^6^ However, Lamb et al described lower VHS values in female dogs than in male dogs.^10^ It should be noted, however, that the difference between males and females in Lamb et al study has been observed in the general population and not for each breed.^10^ In the present study, VHS values of male and female dogs were not significantly different. A possible explanation could be that in our CKCS population there is no sexual dimorphism between male and females, as reported by breed standard (https://www.enci.it/media/2405/136.pdf),^38^ whereas other studies report more variation in this breed.^39^ Finally, no correlation was found between the VHS, BCS, and body weight. This result is in accordance with previous studies in Spitzs, mixed breeds, and Labrador Retrievers,^17^ but not with the results found in Lhasa Apsos and Norwich Terriers.^16^, ^20^ Disagreement between studies may be explained by possible variations in the amount of pericardial fat in different breeds.^18^

The VLAS found in our study population is lower than the values proposed by Malcolm et al and more similar to those reported by Puccinelli et al.^7^, ^21^ The same difference from data reported by literature can be observed for M‐VLAS and RLAD.^8^, ^9^ It is interesting to report that the control group (consisted of healthy subjects) from which normal values of VLAS and M‐VLAS were derived, were composed of only 15 and six dogs, respectively, with only one healthy CKCS included in Malcolm et al study and no CKCS in Lam et al study.^7^ Furthermore, the control group in the study of Salguero et al (RLAD) included only one healthy CKCS. Thus, the presence of other breeds could have raised the proposed VLAS and M‐VLAS ranges and RLAD.

We must also emphasize that all the published data about VLAS, M‐VLAS, and RLAD reported a cut off able to discriminate among subject with or without left atrial enlargement and not the normal radiographic size of the left atrium. It is therefore likely that values obtained in our study are lower for this reason. Further studies including a larger population of healthy and affected by MMVD at different stages CKCS are needed to better clarify the normal reference interval of the VLAS, M‐VLAS, and RLAD in this breed and a cut‐off value useful for discriminate left atrial enlargement. In addition, breed‐specific differences in the VLAS, M‐VLAS, and RLAD, like the VHS, should be considered.

The main limitation for the current study was the small sample size. A larger population could have led to possible differences in the proposed reference range because the CKCS breed is very inhomogeneous in terms of size and morphotype. Body weight in CKCS may vary with different genetic lineages; however, our range of body weight was wide (5‐12 kg), and no correlation was found between the VHS and body weight. Thus, it is reasonable to believe that even increasing the sample population, comparable results would be found. In addition, most previous studies reporting breed‐specific reference values of the VHS included a maximum of 30 cases per breed, as the present study.^10^, ^11^, ^15^, ^16^, ^17^, ^19^ Also like previous studies on radiographic vertebral‐based measurement,^7^, ^16^, ^41^, ^42^ our sample population did not include dogs younger than 12 months.

In conclusion, results of this study support previous studies indicating that breed‐specific reference values for the VHS are needed. Furthermore (as underlined by 2019 ACVIM MMVD guidelines) VHS, VLAS, M‐VLAS, and RLAD breed‐specific reference values should be introduced in the evaluation of thoracic radiographs, also in healthy subjects. In CKCS, the VLAS, M‐VLAS, and RLAD values found in this study can be used as references to avoid misinterpretation of cardiomegaly in this breed. Further studies evaluating the VLAS, M‐VLAS, and RLAD in different canine breeds are warranted.

## LIST OF AUTHOR CONTRIBUTIONS

### Category 1

(a) Conception and Design: Locatelli, Bagardi, Brambilla
(b) Analysis of Data: Bagardi, Manfredi, Bassi, Spediacci, Ghilardi, Zani
(c) Interpretation of Data: Locatelli, Bagardi

### Category 2

(a) Drafting the Article: Bagardi, Locatelli, Manfredi, Bassi
(b) Revising It Critically for Important Intellectual Content: Bagardi, Locatelli, Manfredi, Spediacci, Ghilardi, Zani, Brambilla

### Category 3

(a) Final Approval of the Version to be Published: Bagardi, Locatelli, Manfredi, Bassi, Spediacci, Ghilardi, Zani, Brambilla

### Category 4

(a) Agreement to be Accountable for All Aspects of the Work in Ensuring that Questions Related to the Accuracy or Integrity of Any Part of the Work are Appropriately Investigated and Resolved: Bagardi, Locatelli, Manfredi, Bassi, Spediacci, Ghilardi, Zani, Brambilla

## CONFLICT OF INTEREST

The authors declare that there were no conflicts of interest
